# Protocol for Probing Regulated Lysosomal Activity and Function in Living Cells

**DOI:** 10.1016/j.xpro.2020.100132

**Published:** 2020-10-17

**Authors:** L.V. Albrecht, N. Tejeda-Muñoz, E.M. De Robertis

**Affiliations:** 1 Department of Biological Chemistry, David Geffen School of Medicine, University of California, Los Angeles 90095-1662, USA

## Abstract

Lysosomes are the catabolic center of the cell. Limitations of many lysosomal tracers include low specificity and lack of reliable physiological readouts for changes in growth factor-regulated lysosomal activity. The imaging-based protocols described here provide insights at the cellular level to quantify functions essential to lysosomal biology, including β-glucosidase enzymatic cleavage, active Cathepsin D, and pH regulation in real time. These optimized protocols, applied in different cell types and pathophysiologic contexts, provide useful tools to study lysosome function in cultured living cells.

For complete details on the use and execution of this protocol, please refer to Albrecht et al. (2020).

## Highlights

• Protocol for visualizing and quantitively examining lysosomal catabolism
• Details how organelle pH gradient shifts can be dynamically traced in living cells
• Describes approaches to determine levels of active Cathepsin D in lysosomes
• β-Glucosidase, a target in Gaucher disease, can be assessed by substrate cleavage

## Graphical Abstract

**Figure:**
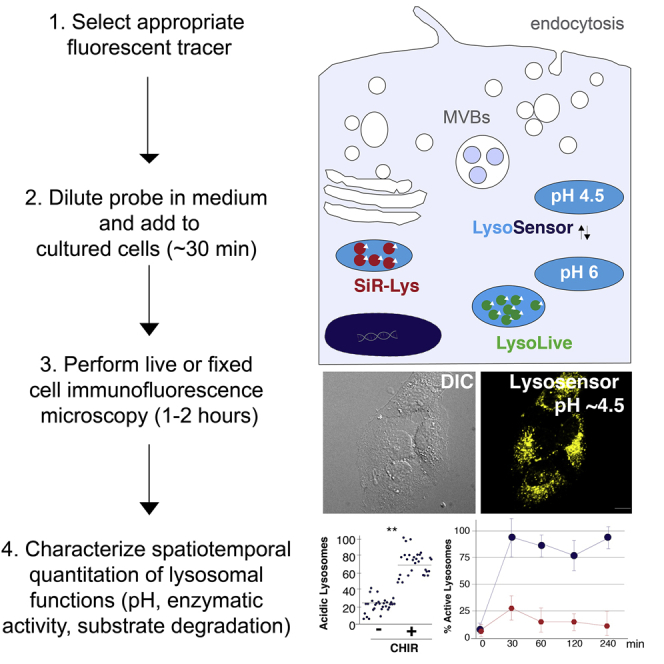

## Before You Begin

Lysosomes lie at the crux of a diverse set of diseases (Ballabio and Bonifacino, 2020; Holland et al., 2020; Lawrence and Zoncu, 2019). Recent work highlights a central role for lysosomal catabolic activity in cancer by restoring cellular building blocks and promoting cell growth. Here, we provide simple assays to measure regulated changes of lysosomal function in living cells such as (1) cleavage of a β-Glucosidase fluorogenic substrate (Harlan et al., 2016) (β-glucosidase is the enzyme mutated in Gaucher disease, the most frequent lysosomal storage disease), (2) lysosomal Cathepsin D (using a pepstatin A peptide probe that binds only to the active form of this protease) (Hartsuck, 1976), and (3) lysosome acidification using the ratiometric pH Yellow/Blue LysoSensor dye. Importantly, rapid cellular and metabolic changes can be followed by adding cell-permeable fluorescent reagents to the culture medium without the need for genetic manipulation or overexpression. These protocols have nuances that are carefully described here to help facilitate the application of these reagents to different systems, cell types, and disease models. In sum, these protocols on lysosomal regulation will be useful for studies on regulated protein degradation, cellular metabolism, and cancer (see Albrecht et al., 2020).

### Maintenance of Cell Culture

**Timing: 2 days**

Lysosomes are present in all animal cells with the exception of mature erythrocytes. Thus, we anticipate that these protocols will be useful for studies in many different cell types. The protocols here were optimized for cell lines that are responsive to growth factor signaling including transformed human lines, Alexander hepatocellular carcinoma (HCC) cells, colorectal carcinoma (SW480) cells, epidermal keratinocyte (HaCaT) cells, cervical adenocarcinoma (HeLa) cells, and Human Embryonic Kidney (HEK293) cells, in addition to monkey kidney (Cos7) cells and mouse fibroblast (NIH-3T3) cells (Albrecht et al. 2020).

***Note:*** Cell line identity tests are being implemented as per requirements for grant proposals and manuscript submissions and should be considered for lysosomal studies. ATCC cell line authentication services can be used to confirm identity of cultured cell types.

1. Culture cells at 37°C in 5% CO_2_ atmosphere in appropriate medium. In our studies, cells are cultured in 10-cm dishes with 10 mL of medium per dish.
  ***Note:*** Normoxia versus hypoxia can affect lysosomal physiology. The current protocol uses ambient oxygen conditions for culturing cells (18 kPa). However, one should also note that growing cells in hypoxic conditions has been reported to impact autophagy and lysosomal activity in addition to the enzymatic activity of cathepsin enzymes (Nagaraj et. al., 2007; Lai et. al., 2016; Hong et. al. 2019).
  a. Frequently used for media include Roswell Park Memorial Institute (RPMI) 1640 or Dulbecco’s Modified Eagle’s Medium (DMEM) supplemented with 10% fetal bovine serum (FBS), glutamine, and antibiotics. As cell line growth conditions vary, we suggest referring to the recipes suggested by the provider source.
    ***Note:*** Cell lines should be tested for mycoplasma contamination as this can alter lysosomal function.
2. For all of the following lysosomal assays, 24 h prior to experimentation cells are lifted from cell culture plate using standard trypsinization (0.05%) cleavage and plated for either live or fixed cell imaging.
  a. We recommend using the advised trypsinization times suggested by the provider for each different cell line.
3. For fixed cell imaging: cells are plated on round coverslips (15 mm) autoclaved or sterilized with ethanol in 12-well dishes to a confluency of 30%–60% Troubleshooting 1
  a. Coverslips stored at 20°C should be sterilized before use by dipping coverslips into ethanol in the tissue culture hood. Let the ethanol evaporate and place one coverslip into one individual well of a 12-well plate. An acid-wash was not used in our protocol.
  b. Cells are plated onto coverslips in the 12-well plate with a volume of 1 mL of medium per well.
  c. Let cells attach to coverslips for 24 h prior to experimentation at 37°C in 5% CO_2_ atmosphere.
4. For live-cell imaging: cells are plated onto 8-well glass-bottom chamber slides with 0.5 mL of medium per well.
  a. Let cells attach to coverslips for 24 h prior to experimentation at 37°C in 5% CO_2_ atmosphere.
  ***Optional:*** Use of fibronectin or collagen coated coverslips can be used to help cells adhere to the glass and help cell spreading for easier image analyses of specific cell lines such as HEK293 cells. Sterile tissue culture-grade fibronectin is incubated with coverslips following ethanol sterilization at a concentration of 100 μg/mL (500 μL per well) for 30 min at room temperature. Coverslips are then washed with PBS three times and once with cell culture medium, prior to seeding.

## Key Resources Table

**Table:** 

REAGENT or RESOURCE	SOURCE	IDENTIFIER
**Chemicals, Peptides, and Recombinant Proteins**		

LysoLive (β-glucosidase substrate)	Marker Gene Tech	Cat# M27745
SiR-Lysosome	Cytoskeleton Inc	Cat# CYSC012
Lysosensor Yellow/Blue DND-160 (PDMPO)	ThermoFisher	Cat# L7545
Tetramethylrhodamine Dextran (TMR-Dx) 70,000 kDa	ThermoFisher	Cat# D1818
Bovine Serum Albumin DeQuenched (BSA-DQ)	ThermoFisher	Cat# D12051
Ovalbumin DeQuenched (DQ)	ThermoFisher	Cat# D12053
Lysotracker Deep Red	ThermoFisher	Cat# L12492
Alexa Fluor 488 Phalloidin	ThermoFisher	Cat# A12379
Cycloheximide	Sigma	Cat# C-7698
Lithium chloride (LiCl)	Sigma	Cat# L4408
CHIR99021	Sigma	Cat# SML1046
DMSO	Sigma	Cat# W387520
5-(N-Ethyl-N-isopropyl) amiloride (EIPA)	Sigma	Cat# A3085
IPA-3	Sigma	Cat# I2285
Sodium chloride (NaCl)	Sigma	Cat# S9888
Wnt3a	Peprotech	Cat# 315-20
Lipofectamine 3000	ThermoFisher	Cat# L3000001
Prolong Antifade	ThermoFisher	Cat#P36930
Prolong Antifade + DAPI	ThermoFisher	Cat# P36931
DMEM	ThermoFisher	Cat#11965092
RPMI 1640	ThermoFisher	Cat#12633012
Glutamine	ThermoFisher	Cat#25030081
Fetal Bovine Serum (FBS)	ThermoFisher	Cat#16000044
Bovine Serum Albumin (BSA)	ThermoFisher	Cat#9048468
Goat Serum	ThermoFisher	Cat#50197Z
Pen-Strep antibiotics	ThermoFisher	Cat#15140122
Triton X-100	ThermoFisher	Cat#HFH10
Fibronectin	ThermoFisher	Cat#33016015
Collagen	Sigma	Cat#C7661
10-cm dish	ThermoFisher	Cat#174903
12-well dish	ThermoFisher	Cat#150628
Circular coverslips	ThermoFisher	Cat# 12545100
8-well glass-bottom chamber slides	ibidi	Cat #80827

**Experimental Models: Cell Lines**		

Alexander hepatocellular carcinoma	ATCC	RRID:CVCL_0485
Cos7	ATCC	RRID:CVCL_0224
HaCaT	ATCC	RRID:CVCL_3300
HeLa	ATCC	RRID:CVCL_0030
HEK293	ATCC	RRID:CVCL_0045
NIH-3T3	ATCC	RRID:CVCL_0594
SW480	ATCC	RRID:CVCL_0546

**Recombinant DNA**		

LifeAct	IMSR	RRID:IMSR_EM:12427
pCS2-mGFP	Addgene	RRID:Addgene_14757

**Software and Algorithms**		

Fiji imaging software	NIH	http://fiji.sc/
MaxEntropy PlugIn	NIH	http://imagej.nih.gov/ij/
Prism 7	GraphPad	http://www.graphpad.com/scientific-software/prism/
Imaris	Oxford	http://imaris.oxinst.com
Zen 2.3 imaging software	Zeiss	http://zeiss.com
Other		
Axio Observer Z1 Inverted Microscope with Apotome	Zeiss	n/a

## Materials and Equipment

### Preparation of Lysosomal Reagents Prior to Use

***Note:*** For all of the following reagents, limit light exposure throughout use.

**Table:** 

Reagent	Traces	[Working]	Kit Contents	No. of Exp. / Kit	λabs (nm)	λem (nm)	Probe
LysoLive (5 mM)	β-Glucosidase substrate	5–20 μM	5 × 10 μL probe 2 × 30 μL Buff.	96 coverslips	490	520	Intensiometric
SiR-Lysosome (1 mM)	Cathepsin D	1 μM	50 nmol probe 1 μmol verapamil	200 coverslips	652	674	Intensiometric
LysoSensor Dye (1 mM)	pH shifts	1–200 μM	20 vials of 50 μL	>1,000 coverslips	329, 384	440, 540	Ratiometric
Lysotracker Deep Red (1 mM)	acidic pH	50 nM	20 vials of 50 μL	>1,000 coverslips	647	668	Intensiometric

1. LysoLive β-Glucosidase substrate
  a. Product arrives at a stock concentration of 5 mM. Opti-Klear Live Imaging Buffer (1×) is also included.
  b. To avoid freeze-thaw: seal in small aliquots of 2–5 μL and when stored at −20°C can remain stable for at least four months.
2. SiR-Lysosome
  a. For 1 mM stock concentration: Dissolve SiR-Lysosome vial in 50 μL anhydrous DMSO.
  b. Compound is unaffected by freeze-thaw. Do not aliquot as this will increase decay. Store SiR-Lysosome at −20°C until use, product is stable for at least three months.
3. LysoSensor
  a. Product arrives as a stock concentration of 1 mM in 50 μL anhydrous DMSO.
  b. To avoid freeze-thaw: seal in small aliquots and store at −20°C.

Equipment setup for analyzing these reagents in cells and tissues can include imaging with widefield or confocal microscopes and with Structured Illumination Microscopy (SIM) or Stimulated Emission Depletion (STED) microscopy in fixed cells. These fluorescent tracers are also suitable for monitoring rapid regulated changes in living cells that can be captured by live-cell imaging.

Here, we performed live imaging using a Zeiss Observer.Z.1 inverted microscope equipped with Apotome.2, an automated scanning stage, the Zeiss PEECON stage top incubation for Temperature/CO_2_ for cell culture. Software analyses can be performed across an array of platforms including Imaris or the publicly available and free resource, Image J. Statistical analyses of quantification are best suited for Prism software.

## Step-by-Step Method Details

This protocol is divided into three main sections that will each offer a unique readout for monitoring lysosomal function.

### LysoLive Assay for Measuring β-Glucosidase Activity *In Vivo*

**Timing: 1–2 h**

**Figure 1:**
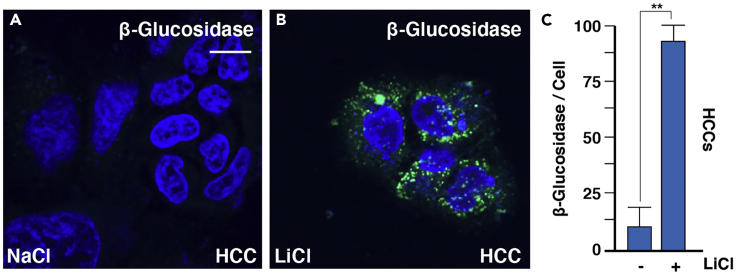
LysoLive Traces β-Glucosidase Activity Where Enzyme Substrate Fluoresces upon Degradation in Lysosomes of Living Cells (A–C) Images above depict representative example of regulated-lysosomal activity in Hepatocellular Carcinoma HCC cells, which can be quantified using ImageJ and the protocols described here. Error bars denote SEM (n ≥ 3) (∗∗p>0.01). Scale bar is 10 μm.

This assay provides a quantitative measure of the activity of a lysosomal enzyme, β-Glucosidase, which is involved in diseases such as Gaucher’s and Parkinsons. LysoLive uses a sensitive substrate of β-glucosidase, GlucGreen, that becomes fluorescent upon enzymatic cleavage proportional to enzyme activity in a living cell (Harlan et al., 2016) (Figure 1.).

1. On the day of the experiment, prepare staining medium by diluting the β-glucosidase substrate (stock concentration of 5 mM) to a working concentration of between 5 – 20 μM in DMEM with or without 10% FBS, in the absence of antibiotics. We recommend starting with the smallest concentration of probe necessary of 5 μM (0.5 μL probe per 500 μL DMEM). Troubleshooting 2
  ***Note:*** In deciding whether to use FBS in these assays, one should be aware that serum starvation is frequently applied to decrease effects that could be exerted by growth factors in the serum. Additionally, serum starvation can serve as a way to synchronize cell cycle phases of plated cell populations as lysosomal activity may change with the cell cycle. As this can increase autophagy, we include FBS (10%) in most of our studies. If serum starvation is performed, cells should be incubated in serum free medium for at least 4 h prior to experimentation.
2. For fixed cell imaging: use cells plated in a 12-well dish (30%–60% confluency) with one coverslip per well.
  a. Incubate cells with β-Glucosidase substrate in DMEM for 20 min at 37°C in 5% CO_2_ atmosphere (500 μL per well). Troubleshooting 3
  b. Wash 3 times with ice-cold PBS (pre-made from Sigma) to remove probe (0.5 mL per well).
  ***Note:*** Ice-cold PBS washes should be performed gently to not disturb or displace the plated cells and rapidly to decrease time between removing the substrate and cell fixation.
  c. Fix cells for 15 min in 4% PFA diluted in PBS (0.5 mL per well) at 20°C with limited light exposure. Troubleshooting 4
  **Pause Point:** Cells can be left in PBS at 4°C following fixation with limited light exposure for 16 h.
  d. Wash with PBS three times to remove PFA (0.5 mL per well).
  ***Optional:*** Block cells for 1 h in blocking buffer containing 5% goat serum, 5% bovine serum albumin, PBS, sodium azide to reduce background from nonspecific binding.
  e. Mount cells in mounting media with DAPI onto a standard mounting glass slide. Make sure that the microscope slide is clean of fingerprints or dust.
  f. Apply mounting medium with DAPI onto glass microscope slide using 20 μL per each coverslip. Check that there are no air bubbles in the pipette tip and lay down coverslip onto the mounting medium slowly to avoid air bubbles from forming.
  g. Dry in dark area at 20°C for 16 h prior to imaging.
  h. After coverslips have dried they should be stored at 4°C where staining remains at its optimal intensity for at least two weeks.
3. For live-cell imaging, use cells plated in an 8-well glass-bottom chamber slides (30%–60% confluency):
  a. Incubate cells with β-Glucosidase substrate in DMEM for 20 min at 37°C in 5% CO_2_ atmosphere (500 μL per well).
  b. Wash with ice-cold PBS three times to remove the probe (0.5 mL per well).
  c. Image cells for in live-cell imaging chamber with temperature and cell culture conditions of 37°C in 5% CO_2_ atmosphere.

### SiR-Lysosome Assay for Measuring Active Lysosomal Cathepsin D

**Timing: 1–2 h**

**Figure 2:**
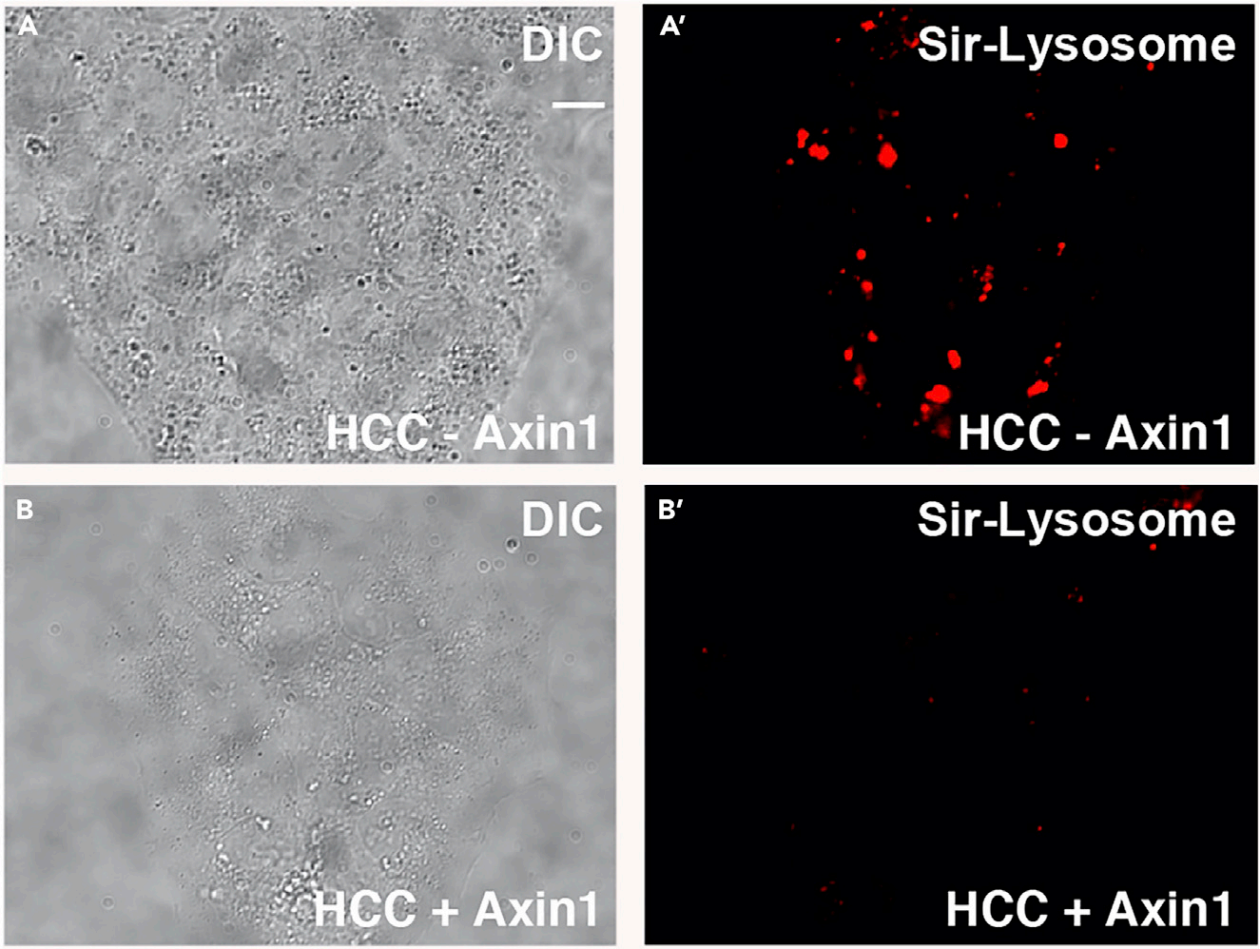
SiR-Lysosome Traces Cathepsin D Activity in Living Cells and Can Be Monitored by Live-Cell Imaging (A and B′) Images here are stills of a movie taken of SiR-Lysosome in HCC cells. Left images are in DIC while right images are from the red fluorescence filter. Images were captured with an exposure time of 500 ms and a light intensity of 95%. See corresponding Methods Video S1. Scale bar, 10 μm.

This assay step monitors changes in the amount of the active form of lysosomal Cathepsin D protease (Figure 2.). SiR-lysosome is a cell-permeable peptide with the sequence of pepstatin A that is known to bind specifically to active Cathepsin D and has been conjugated to a fluorescent photostable silicon rhodamine (SiR) dye. Importantly, its far-red emission (imaged in standard Cy5 filter sets) decreases phototoxicity and autofluorescence with compatibility for GFP- and mCherry-tagged proteins.

1. Dilute SiR-Lysosome (1 mM stock concentration) to a working concentration of 1 μM in DMEM (0.5 μL probe per 500 μL DMEM), with or without 10% FBS in the absence of antibiotics.
  ***Optional:*** Addition of the calcium channel blocker, verapamil (working concentration of 1 μM), to SiR-Lysosome DMEM solution can enhance signal intensity. This reagent is provided with the kit, as noted in the Table above. Final concentrations of DMSO should not exceed 2%.
2. For fixed cell imaging: Use cells plated in a 12-well dish (30%–60% confluency) with one coverslip per well.
  **Pause Point:**
  a. Incubate cells with SiR-Lysosome in DMEM for 5 – 60 min at 37°C in 5% CO_2_ atmosphere (500 μL per well).
  b. Wash with ice-cold PBS three times to remove the probe (0.5 mL per well).
  c. Fix cells for 15 min using 4% PFA diluted in PBS at 20°C with limited light exposure (0.5 mL per well).
  d. Wash with PBS three times to remove PFA (0.5 mL per well).
  e. Mount cells in mounting media with DAPI onto a standard mounting glass slide. Make sure that the microscope slide is clean of fingerprints or dust.
  f. Apply mounting medium with DAPI onto glass microscope slide using 20 μL per coverslip. Check that there are no air bubbles in the pipette tip and lay down coverslip onto the mounting medium slowly to avoid air bubbles from forming.
  g. Dry in dark area at 20°C for 16 h prior to imaging.
  h. After coverslips have dried, they should be stored at 4°C where staining does not fade for at least two weeks.
    **Pause Point:**
3. For live-cell analyses (as seen in Methods Video S1): Use cells plated in a 8-well chamber (30%–60% confluency).
  a. Incubate cells with 1 μM SiR-Lysosome in DMEM for 5 – 60 min at 37°C in 5% CO_2_ atmosphere (500 μL per well).
  b. Wash with ice-cold PBS three times to remove the probe (0.5 mL per well).
  c. Insert cell chamber for live imaging with conditions of 37°C in 5% CO_2_
  d. Image cells for 1 h where with an image acquisition rates of 1 frame per 30 s. Live-cell imaging microscopy approaches detailed below.
  e. Cell viability can be maintained for 12 h, depending on cell type.

Methods Video S1. SiR-Lysosome (Far Red) Traces the Enzymatic Activity of Cathepsin D in Lysosomes, Related to Figure 2 Duration of time-lapse film was 30 min and is related to Figure 2. Alexander hepatocellular carcinoma (HCC) cells reconstituted with Axin1 protein were incubated for 1 h with Sir-Lysosome (1 μM). Images were collected in DIC and red fluorescence filter, acquired every 30 s, and images were acquired using the Zeiss Observer.Z.1 inverted microscope with Apotome.2. Fluorescence filters were controlled by Axiovision 4.8 software.

### LysoSensor Yellow-Blue Assay for Measuring Physiological pH Shifts

**Timing: 1–2 h**

**Figure 3:**
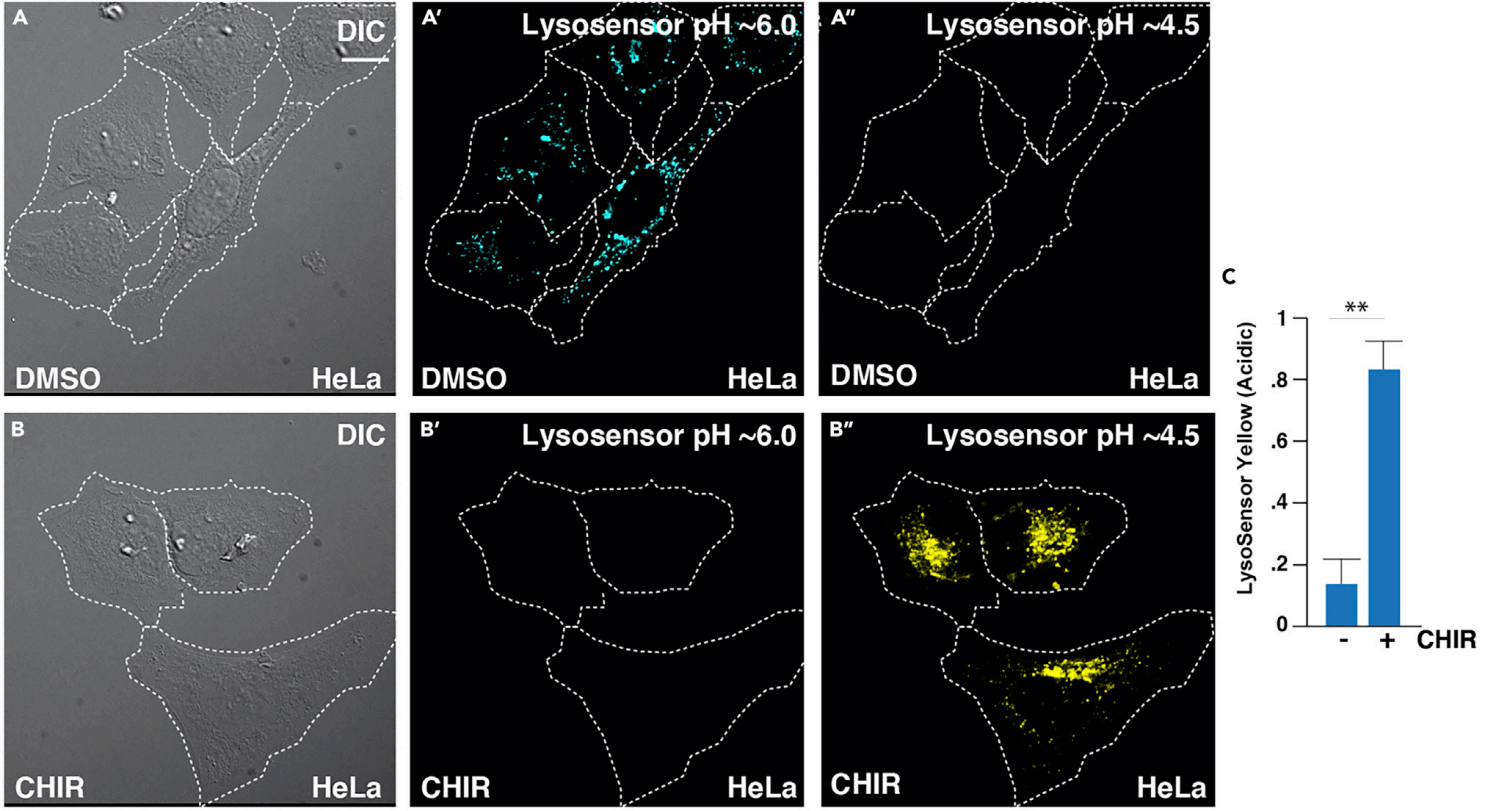
LysoSensor Traces Lysosomal pH Gradients in Living Cells (A–C) Images depicted here demonstrate an increase in lysosomal activity visualized as increased yellow fluorescence following treatment with GSK3 inhibitor, CHIR, in HeLa cells. Yellow fluorescence can be quantified as a marker of lysosomal activity. Error bars denote SEM (n ≥ 3) (∗∗p > 0.01). Scale bar, 10 μm.

This step accomplishes a way to monitor changes in lysosomal pH in living cells (Figure 3.). pH is a fundamental aspect of lysosomal biology and a central target for drug design (Diwu et al., 1999). As one example, chloroquine operates as a mechanism to decrease the lysosomal acidity essential for enzymatic activity. Similar to the commonly used Lysotracker reagent, LysoSensor Yellow/Blue DND-160 is an acidotropic probe that accumulates in acidic lysosomes following protonation. However, while Lysotracker only marks acidic organelles, LysoSensor has the additional advantage of tracking fluctuations in pH. LysoSensor serves as a ratiometric marker of lysosomal pH (pK_a_ of ~4.2) as it is conjugated to the compound, 2-(4-pyridyl)-5-((4-(2-dimethylaminoethylaminocarbamoyl)-methoxy)-phenyl)oxazole (PDMPO) that exhibits pH-dependent dual-excitation and dual-emission spectral peaks. Thus, LysoSensor produces a blue fluorescence in weakly acidic organelles and shifts to yellow in more acidic lysosomes (Albrecht et al., 2020). Additional advantages including high photostability of PDMPO fluorescence facilitate image-based assays.

1. Dilute LysoSensor (stock concentration of 1 mM) to a working concentration in the range of 1 – 200 μM in DMEM (for 1 μM use 0.5 μL probe per 500 μL DMEM) with or without 10% FBS in the absence of antibiotics. LysoSensor aliquots can be thawed at room temperature.
  ***Note:*** We recommend starting with probe concentration of 1 μM to reduce artifacts of overloading, which can appear as nonspecific binding of the probe throughout the cell. This can be assessed by co-labeling cells with Lysotracker Deep Red, which should colocalize with acidic lysosomal vesicles. For Lysotracker co-staining, probes should be used as described here with the addition of 50 nM Lysotracker, from a stock concentration of 1 mM, to DMEM (2.5 nL probe per 500 μL DMEM) with LysoSensor probe.
2. For fixed cell imaging: use cells plated in a 12-well dish (30%–60% confluency) with one coverslip per well.
  a. Incubate cells with LysoSensor in DMEM for 5 min at 37°C in 5% CO_2_ atmosphere (500 μL per well). Troubleshooting 4
    ***Optional:*** Lysotracker Far Red, which only marks the acidic lysosomes can be performed side-by-side, as a positive control for confirming pH gradient shifts. While PFA fixation is typically used for lysosomal studies using standard probes, we recommend that comparing the pH changes of fixed cells with those seen by live-cell imaging could provide a powerful method to directly determine whether fixation conditions impact pH shifts within individual experiments. pH calibration curves should be performed prior to imaging, as described previously (Diwu et al., 1999).
  b. Wash with ice-cold PBS three times to remove the probe (0.5 mL per well).
    Fix cells for 15 min using 4% PFA diluted in PBS at r20°C with limited light exposure (0.5 mL per well).
  c. Wash with PBS three times to remove PFA (0.5 mL per well).
  d. Mount cells in mounting media (without DAPI) onto a standard mounting glass slide. Make sure that the microscope slide is clean of fingerprints or dust.
  e. Apply mounting medium (without DAPI) onto glass microscope slide using 20 μL per each coverslip. Check that there are no air bubbles in the pipette tip and lay down coverslip onto the mounting medium slowly to avoid air bubbles from forming.
  f. Dry in dark area at 20°C for 16 h prior to imaging.
  g. After coverslips have dried, they should be stored at 4°C where staining does not fade for at least two weeks.
    **Pause Point:**
3. For live-cell analyses: use cells plated in an 8-well glass-bottom chamber (30%–60% confluency).
  a. Incubate cells with LysoSensor in DMEM for 5 min at 37°C in 5% CO_2_ atmosphere (500 μL per well).
  b. Wash with ice-cold PBS three times to remove the probe (0.5 mL per well).
  c. Insert cell chamber for live imaging with conditions of 37°C in 5% CO_2._
  d. Image cells for 1 h where with an image acquisition rates of 1 frame per 30 s. Live-cell imaging microscopy approaches detailed below.
  e. Cell viability can be maintained for 12 h, depending on cell type.

### Drug Treatments or Growth Factor Signaling Studies (Optional Steps)

**Timing: 2–4 h or 24 h**

The protocols described above will be particularly useful for studies of lysosomal regulation by tumor suppressors (such as Adenomatous Polyposis Coli or Axin1), oncogenes (such as activated Ras mutations) or following treatment with growth factors (such as Wnt or EGF). As one example, canonical Wnt signaling, a pathway implicated in many cancers (Nusse and Clevers, 2017), was found to be a key regulator of lysosomal function. These studies uncovered that lysosome function is normally repressed by GSK3 via the tumor suppressor Axin1 (Albrecht et al., 2020). Endocytosed Wnt receptor complexes result in the sequestration of GSK3 and Axin1 from the cytosol inside membrane-bounded multivesicular bodies (MVBs)/lysosomes through the membrane invagination process known as microautophagy (Taelman et. al., 2010; Vinyoles et al., 2014). Significant increases in lysosomal activity followed activation of the Wnt pathway initiated by short treatments with recombinant Wnt3a growth factor protein or pharmacological GSK3 inhibitors and in cells with mutations in the tumor suppressor protein Axin1. Within minutes of Wnt stimulation, the cortical actin machinery is activated and results in typical plasma membrane ruffling associated with macropinocytosis (Albrecht et al, 2020) and endocytosis of Bovine Serum Albumin (Albrecht et al., 2018), both through β-catenin dependent and independent mechanisms (Redelman-Sidi et al., 2018; Tejeda-Muñoz et al., 2019). GSK3 inhibition (or Wnt3a) triggered macropinocytosis within minutes, resulting in a dramatic increase in lysosomal activity within 20 min or less, even in the absence of new protein synthesis.

While optimal concentrations and timeframes will need to be determined for different cell types and physiologic contexts, the following steps outline protocols for studies of Wnt signaling as a reference starting point for studies on regulated lysosome activity.

1. 24 h following cell plating onto coverslips before/during incubation with lysosomal tracers:
  a. Drug treatments: GSK3 inhibitors (40 mM LiCl or 8 nM CHIR99021) or controls (40 mM NaCl or DMSO) can be added a few minutes prior or together with medium plus lysosomal tracers (LysoLive, Sir-Lys, LysoSensor).
  b. Growth factor treatments: Wnt3a (100 ng/mL) and control buffer in DMEM can be added together with lysosomal tracer, according to the probe recommendations above. Total time with Wnt3a was typically 20 min.
  c. Protein synthesis inhibition: cycloheximide (20 μg/mL) should be added to cells at least 4 h prior to incubation with DMEM + lysosomal tracer.
  d. Transfection: 24 h prior to lysosomal assays, the desired DNA transfections can be tested as is standard following lipofectamine 2000 manufacturer protocols.
2. Additional insight into regulated lysosomal function in relation to endocytosis
  a. To study extracellular protein degradation in lysosomes with lysosomal activity tracers: dilute Bovine Serum Albumin DeQuenched (BSA-DQ) (5 μg/mL), or Ovalbumin-DQ (5 μg/mL) in DMEM and incubate with cells for 30 min. BSA-FITC (5 μg/mL) can be used as a fluid-phase marker of endocytosis that fluoresces without requiring lysosomal degradation (Albrecht et al., 2018 and 2020).
  b. To study specifically macropinocytosis with lysosomal tracers: dilute TMR-Dextran 70 kDa (1 mg/mL) in culture medium and incubate with cells for 30 min or less.
3. Proceed with 3 times PBS washes and fixation in 4% PFA diluted in PBS (0.5 mL per well), as above.
4. For antibody co-staining:
  a. Wash with PBS three times to remove PFA (0.5 mL per well).
  b. Permeabilize cells with either digitonin (6.5 μg/mL) or Triton X-100 (0.15%) diluted in PBS with 0.5 mL per well).
  c. Wash with PBS three times to remove detergent (0.5 mL per well).
  d. Block cells for 1 h in blocking buffer containing 5% goat serum, 5% bovine serum albumin, PBS, and sodium azide (0.5 mL per well).
  e. Primary antibodies diluted in blocking buffer should be incubated with cells for 1 h at 20°C with limited light exposure (0.5 mL per well).
    ***Note:*** The use of antibody staining with lysosomal proteins such as Lysosome Associated Membrane Protein 1 (LAMP1) with standard fluorescent lysosomal markers is routinely performed to confirm lysosome localization and could be applied with any of the reagents described here (Klionsky et. al., 2008).
  f. Wash in 3 times with PBS (0.5 mL per well).
  g. Secondary antibodies diluted in blocking buffer should be incubated with cells for 40 min at 20°C (0.5 mL per well).
  h. Wash 3 times with PBS (0.5 mL per well).
  i. Mount cells in mounting media without DAPI.
  j. Dry in dark area at 20°C for 16 h prior to imaging.
    **Pause Point:**

## Expected Outcomes

Besides Wnt, there are other growth factors such as EGF (Haigler and Cohen, 1979) and mutations in the RTK-Ras-PTEN-PI3K-Rac-Pak1 axis pathway in cancer (Commisso et al, 2014; Hodakoski et al., 2019) that increase macropinocytosis and presumably lysosome activity due to increased membrane trafficking. Thus, the rapid assays for lysosomal activity detailed here could be useful in a variety of cell biological situations. These techniques will provide spatiotemporal measurements with 2D readouts such as the number size and location of active lysosomal organelles, lysosomal pH fluctuations, and rates of lysosomal enzymatic activity.

## Quantification and Statistical Analysis

**Figure 4:**
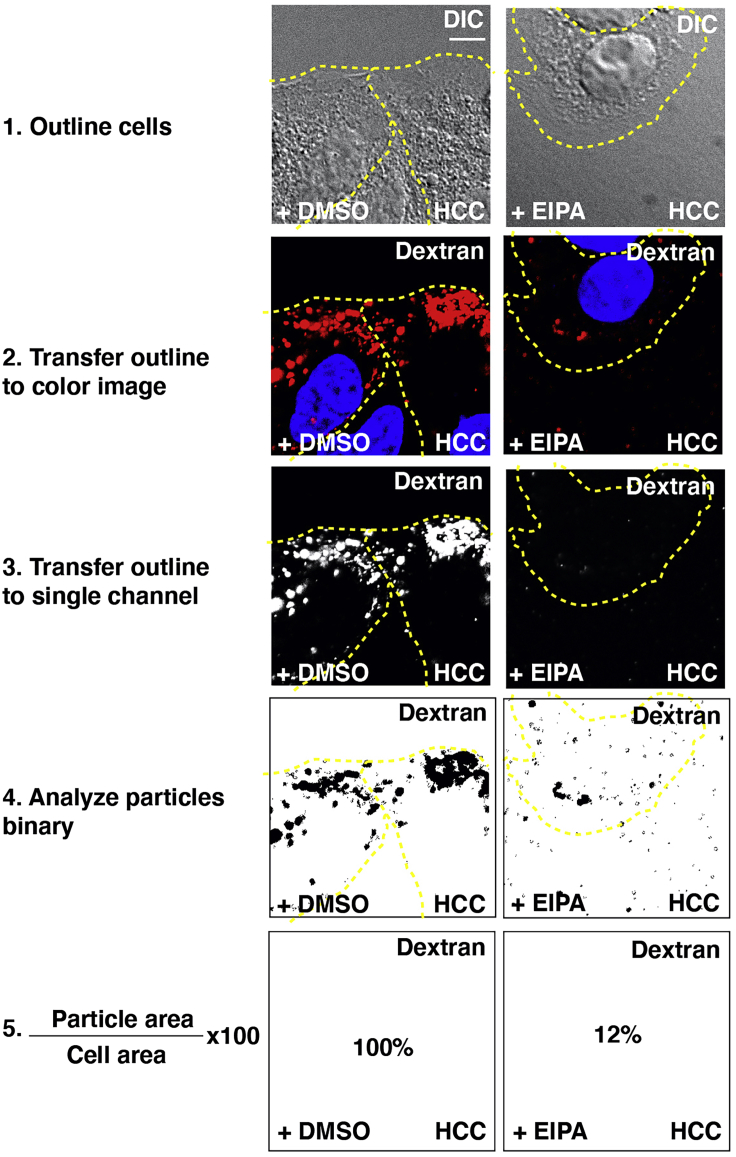
Representative Example of the Steps for Quantifying Fluorescence of Intracellular Organelles Relative to Total Cell Areas Acquire images in both DIC and in fluorescent channels of interest. DAPI (blue) can also be useful as a reference for counting total cell numbers per frame in alternative types of quantification. Right panels contain a pharmaceutical inhibitor and act as a negative control for the tracer of choice. Scale bar, 10 μm.

Data processing of these reagents can include both live and fixed image-based analyses to provide spatiotemporal resolution of rapid lysosomal regulation in living cells. Quantification can be performed using software platforms such as Imaris or ImageJ. Statistical analyses of quantification are best suited for Prism software. Herein, this protocol describes quantification methods using ImageJ (Figure 4.).

1. Studies on fixed cells were performed using a Zeiss Imager.Z1 upright fluorescence microscope with Apotome differential interference contrast (DIC) and fluorescent filters for DAPI, FITC, and rhodamine. Images were acquired using 5×, 10×, 20×, 40× oil, or 63× objectives using the Zeiss Zen software. For live-cell imaging, images were collected with a Zeiss Observer.Z.1 inverted microscope with Apotome, DIC, and Colibri LED with green fluorescence filters using a 63× oil Plan-APOchromat objective. The microscope including the fluorescence filters were controlled by Axiovision 4.8 software. Both microscopes are fully motorized and encoded. Video editing can be performed using the Adobe Premiere Pro CC 2019 software or ImageJ. We recommend using Fiji, which is a distribution of ImageJ that contains image analysis plugins such as Coloc2 and can be downloaded http://fiji.sc/, as described below.
  ***Note:*** Exposure times for each individual experiment were determined first for the brightest sample, avoiding overexposure, and remained constant throughout the imaging procedure. Exposure times may range from 100 to 1,000 ms. Multiple random fields should be captured for each coverslip as this will be used in the following quantifications. If not all images can be captured in one day, slides can be stored in the dark at 4°C for at least two weeks. In addition to the exposure time, the light intensity can also cause overexposure. For our studies, we used the Colibri LED and Zeiss AXIOCAM MRM REV camera with a binning of 1 × 1. For live imaging, cells were imaged and cultured on 8-well glass-bottom chamber slides. Methods Video S1 and Figure 2 highlight stills from a movie of SiR-Lysosome using an exposure of 500 ms and a Colibri light intensity of 95%.
2. Acquired images are saved as .czi, with at least five z-stacks in the Zeiss software program.
3. Load .czi into ImageJ and separate stack of images for each acquired channel.
4. Select one image from your complete experiment (at least 10 images in each condition)
5. Duplicate image (command + D on a Mac or control + shift + D in Windows), as one will be used for further processing.
  ***Optional:*** Background subtraction can be performed in with a rolling ball radius of 5 pixels (Process, Subtract Background)
6. First, threshold image (Image, Adjust, Threshold) and select “Dark Background” and “Auto” or to do this manually set the top sliding bar to right of peak.
7. Take note of threshold values for the reference image, open all images to be used for quantification, and “apply threshold values to all.”
  ***Note:*** Successful thresholding should have all lysosomal organelles marked with a red threshold signal.
8. To determine the relative size of intracellular vesicles relative to total cell volume:
  ***Note:*** Region of interest (ROI) manager is a generally useful function in ImageJ with a variety of applications. For example, ROIs are selected areas that can be saved and also applied to a separate image from a different channel or timepoint.
  a. First, measure the areas of each cell:
    i. Using the differential interference contrast (DIC) image, outline cell regions (using circle or polygon tool) and measure cell area (command + M on a Mac or control + M on Windows).
  b. Next, measure the areas of each lysosome within a cell:
    i. Overlay outlined cells from DIC onto the corresponding fluorescent binary threshold image (Edit, Selection, Restore Selection).
    ii. Using one cell, measure the area of lysosomes (Analyze, Analyze Particles) inclusion criteria for particles include size 0-infinity, circularity 0.00–1.00 with “clear results” and “summarize” selected. Repeat for each cell.
  c. Finally, to assess total lysosome area in relation to total cell area per field:
    i. Copy measured values for lysosome (“particle”) areas (step b) and total cell areas (step a) into Prism.
    ii. Divide the area of lysosomes (step b) by the cell area (step a) and multiply by 100.
    iii. Repeat for each individual cell, from each field, and take the average.
    iv. Once data is incorporated into Prism, perform relevant statistical analyses.
  9. Quantitative ratio measurements of lysosomal pH gradients:
    a. LysoSensor has a blue fluorescence in less acidic organelles and yellow in more acidic lysosomes with dual-emission peaks of 440 and 540 nm. Images from both channels should be acquired in 16-bit.
    b. Image blank regions of the coverslips to correct for background.
    c. Additional controls include a separate coverslip with cells treated with Lysotracker, marking only acidic vesicles, and a coverslip of cells without any tracer.
    d. Open all images for one experiment, open brightness and contrast (command + C on a Mac or control + shift + C in Windows), and set appropriate fluorescence intensity for the brightest image, and “apply to all.” Save TIFF images.
    e. Outline cells as above and measure fluorescence intensity of individual cells in both channels across all samples.
    f. Incorporate data into Prism for relevant statistical analyses.
  10. To determine number of active organelles formed relative to total numbers of formed vesicles:
    ***Note:*** Size measurements for the organelle of interest should be considered before beginning these analyses to rule out vesicles not relevant for the study. For example, only vesicles with diameters greater than 200 nm are selected for macropinocytosis studies.
    a. To quantify the number of vesicles formed:
      i. Set thresholds for DIC images using MaxEntropy in ImageJ from the PlugIn tab, which can be downloaded directly from imagej.nih.gov.
      ii. Distinguish individual vesicles in DIC images using the binary watershed function, which can be found in “Process” tab.
      iii. To count number of vesicles, apply the Particle Function using 0.2–7 μm, circularity –1.
    b. To assess lysosomal activity relative to total number of vesicles formed:
      i. Open corresponding fluorescent images (LysoLive, SiR-Lys, LysoSensor)
      ii. Normalize brightness and intensity within individual channels that were each taken at constant exposure time per channel (command + C on a Mac or control + shift + C in Windows).
      iii. Outline cells as above and measure the fluorescence intensity of individual cells
      iv. Plot fluorescent intensity of an individual cell against the number of vesicles quantified for that cell from the DIC in Prism or Excel.
  11. Colocalization analyses of lysosomal tracer:
    a. Open all images within a single experiment
    b. Normalize brightness and intensity within individual channels that were each taken at constant exposure time per channel (command + C on a Mac or control + shift + C in Windows).
    c. Apply the Coloc 2 to perform either Pearson, Manders, Costes, or Li analyses, which should be determined based on the relevant endpoint for different types of experimental design. This can be found in the Analyze tab, under colocalization (Analyze, Colocalization, Coloc2).
  12. Perform appropriate statistical analyses using Prism
    ***Note:*** Increasing the number of cells used for quantification will also lead to increased accuracy in data interpretation and more statistical significance. In all cases, the total number of cells counted should be between 25–200 cells per condition, in addition to random fields and blank cells, and in triplicate experiments performed on separate days.
    a. Measurements should be included for data from three or more independent experiments, which can be shown as the mean ± SEM. Statistical analyses of computer-assisted particle analyses were performed using two-tailed *t* tests for two-sided comparisons where statistical significance was defined as ∗p < 0.05, ∗∗p < 0.01, ∗∗∗p < 0.001.
    b. Images chosen for display in figures should be representative images for an entire experiment with normalized fluorescent intensity values.
    ***Note:*** Statistical analyses can be performed using Excel. However, Prism has a user-friendly interface for easily quantitating a diverse array of statistical analyses and generates graphs that are easily incorporated into Illustrator Adobe software for assembling figures for publication.

## Limitations

Possible limitations for these assays are intrinsic to the reagents as some tracers when left with the cells for too long can change lysosomal properties that are being measured. For example, the goal of using the LysoSensor is to monitor pH within lysosomes, however, prolonged incubation with lysosomotropic probes can lead to lysosomal alkalization. Problems can also arise as a result of incubation times that are too short, resulting in low fluorescence intensity. Further, live-cell imaging of LysoSensor pH gradient tracer can present difficulties as pH shifts occur rapidly on the order of minutes, which could be missed between the experimental setup and start point of image acquisition. Thus, the troubleshooting guidelines outlined below should be incorporated into use of this protocol to help mitigate some of these risks. Environmental factors such as growth medium, cell type, and drug treatment will require further optimization in independent lab settings. For this reason, the protocols included here describe methods with transparency and maximal details to increase validity of data analyses.

## Troubleshooting

### Problem 1

High background fluorescence or low signal when analyzing cells stained with lysosomal markers.

### Potential Solution

Cell confluency can influence lysosomal activity. Low fluorescence could result from high cell density on coverslips.

### Problem 2

High background fluorescence of β-glucosidase marker in live or fixed cells.

### Potential Solution

Concentrations of β-glucosidase substrate can be optimized between 5 – 100 μM.

### Problem 3

Rapid changes in lysosomal activity can occur within minutes but fluorescence may be too low for detection at these times. Low fluorescence of β-glucosidase marker in live or fixed cells.

### Potential Solution

Time frames of β-glucosidase substrate incubation can be optimized for desired outcome. Recommended times for regulated lysosomal activity changes range between 5 - 240 min.

### Problem 4

Nonspecific artificial staining or high background in fixed cell imaging.

### Potential Solution

Permeabilization with a gentle detergent such as digitonin, or low concentrations of Triton X-100 0.15% (both diluted in PBS) can be used to reduce background fluorescence. This procedure should be performed prior to blocking, where cells are incubated with either 6.5 μg/mL digitonin or 0.15% Triton X-100 with 0.5 mL per coverslip in an individual well of a 12-well dish for 10 min on ice (Albrecht et. al., 2018). Following this, detergent should be removed with three washes of PBS (0.5 mL per well for each wash).

### Problem 4

Low signal intensity of LysoSensor in live or fixed cells.

### Potential Solution

Incubation for long periods with LysoSensor could have the off-target effect of alkalinizing lysosomal pH, short incubation times are therefore very important.

## Resource Availability

### Lead Contact

Further information and requests for resources and reagents should be directed to and will be fulfilled by the Lead Contact, LV Albrecht (lalbrecht@mednet.ucla.edu).

### Materials Availability

No new materials were generated in this study.

### Data and Code Availability

This protocol includes all datasets generated or analyzed during this study and inquiries about data processing can be directed to the Corresponding author.
